# P334: Catalysing local production of alcohol based handrub in african hospitals: the power of south-north-south partnership

**DOI:** 10.1186/2047-2994-2-S1-P334

**Published:** 2013-06-20

**Authors:** L Bengaly, JD Hightower, P Bonnabry, SB Syed

**Affiliations:** 1 Pharmacy, CHU Hospital Gabriel Touré, Bamako, Mali; 2 African Partnerships for Patient Safety, World Health Organization, Harare, Zimbabwe; 3 Pharmacie des HUG, Geneva University Hospitals, Geneva, Switzerland; 4 WHO Patient Safety, World Health Organization, Geneva, Switzerland

## Introduction

The 58^th^ WHO African Regional Committee in 2008 called for action in 12 patient safety action areas, including infection prevention and control (IPC). Hand hygiene through use of alcohol based handrub (ABHR) is a clear mechanism to translate a regional mandate into local action. However, ABHR availability is a key challenge in African hospitals.

## Objectives

To describe how south-north-south partnership mechanisms can be utilized to enhance capacity in the local production of ABHR in African hospitals.

## Methods

On-going activity reports from the WHO African Partnerships for Patient Safety (APPS) were examined to capture key transferable lessons for the global IPC community. The focus of the examination was twofold: mechanism of partnership functioning and potential for replication.

## Results

Four key steps were identified in partnership functioning. First, develop expertise in a hospital in Mali through a north-south partnership with Geneva University Hospitals. Second, develop capacity in APPS partnership hospitals in Senegal, Mali, Cameroon through a south-north-south mechanism. Third, test ABHR production workshop design through capacity-building in multiple hospitals in Yaoundé, Cameroon through a Mali-Cameroon partnership. Fourth, scale-up capacity in 6 further African countries (Ghana, Mozambique, Rwanda, Tanzania, Zambia, Zimbabwe) through a multi-country south-south ABHR production workshop led by Malian expertise and involving APPS hospitals in each country. The end product from this multi-step process is a practical ABHR production workshop with quality control in-built, designed through partnership and available for wide replication in the African Region.

## Conclusion

Calls for patient safety and IPC improvement can be considered hollow in the face of structural challenges in many African hospitals. One emerging solution is the development of country-based capacity in ABHR production through the use of a south-north-south partnership mechanism. The co-designed practical ABHR production workshop can now be utilized for local in-country action on hand hygiene through ABHR production.

## Disclosure of interest

None declared

